# Supplementary material for: Complexome profiling of the Chlamydomonas psb28 mutant reveals TEF5 as an early PSII assembly factor
Source: Plant Cell. 2025 Mar 17;37(6):koaf055. doi: 10.1093/plcell/koaf055 (PMC12164590; doi:10.1093/plcell/koaf055)
Supplement: koaf055_Supplementary_Data [file koaf055_supplementary_data.zip › psb28_interactive.zip!/plots/intensity/qualityPlots/peptides/_Cre01.g019600.t1.2.html]

### Cre01.g019600.t1.2

Trivial name:   
SeqCoverage [median]: 11.40  
Mass: 23.84 kDa  
  
 

| Sequence | MissedCleavage | Mass [Da] | wtIntensity | muIntensity | Score |
| --- | --- | --- | --- | --- | --- |
| MVSNSGVLVGTATVDR | 0 | 1604.82 | 0.00 | 1124.61 | 164.64 |
| TVLTTETTK | 0 | 992.54 | 338.47 | 4119.14 | 70.92 |
|  |  |  | 338.5 | 5243.7 |  |
